# Vaginal microbe-binding antibody interactions with a *Lactobacillus crispatus* live biotherapeutic following treatment for bacterial vaginosis

**DOI:** 10.1128/spectrum.03983-25

**Published:** 2026-09-01

**Authors:** Rachel Liu, Eric Armstrong, Anke Hemmerling, Steve Miller, Sanja Huibner, Laurel Lagenaur, Thomas P. Parks, Craig R. Cohen, Bryan Coburn, Rupert Kaul

**Affiliations:** 1 Department of Medicine, University of Toronto 7938, Toronto, Canada; 2 Toronto General Hospital Research Institute, University Health Network 198139, Toronto, Ontario, Canada; 3 Department of Obstetrics, Gynecology & Reproductive Sciences, University of California, San Francisco, San Francisco, California, USA; 4 Department of Laboratory Medicine, University of California, San Francisco, San Francisco, California, USA; 5 Osel, Mountain View, California, USA; Houston Methodist Hospital, Houston, Texas, USA

**Keywords:** female genital tract, live biotherapeutic, bacterial vaginosis, reproductive health, mucosal antibodies, mucosal immunity

## Abstract

The vaginal application of LACTIN-V, a live biotherapeutic containing *Lactobacillus crispatus* CTV-05, reduces the recurrence of bacterial vaginosis (BV) and promotes *L. crispatus* colonization; however, colonization success is variable. In 65 women with BV randomized to LACTIN-V or placebo following metronidazole treatment, we evaluated whether pre-existing or treatment-induced vaginal *L. crispatus* CTV-05-binding antibodies shaped colonization outcomes. CTV-05-binding IgA and IgG were commonly detected prior to BV treatment, with IgA predominating. Neither total nor CTV-05-binding IgA or IgG predicted colonization resistance; instead, baseline CTV-05-binding IgA was associated with colonization permissiveness. LACTIN-V application did not change overall vaginal antibody concentrations or induce CTV-05-binding IgA or IgG. Overall, *L. crispatus* CTV-05-binding IgA and IgG were common but are not a barrier to CTV-05 colonization, nor induced by LACTIN-V application.

**IMPORTANCE:**

Recent clinical trials show that vaginal live biotherapeutics (therapeutic grade probiotics) containing the “optimal” vaginal species *Lactobacillus crispatus* have the potential to enhance vaginal health. However, application of these live biotherapeutics often does not lead to successful colonization of the vagina by *L. crispatus*. Prior studies have shown that the vagina often contains antibodies that bind *L. crispatus*, and we used samples from a clinical trial of the product Lactin-V to test the hypothesis that such antibodies may be induced by application and prevent colonization. Importantly, we confirmed that these antibodies were frequently found in the vagina of participants before application, but that they did not block (and may even have helped) colonization, and were not induced by the product. This is important information for the growing field of vaginal live biotherapeutics.

**CLINICAL TRIALS:**

This study is registered with ClinicalTrials.gov as NCT02766023.

## INTRODUCTION

Bacterial vaginosis (BV) is a common vaginal condition that affects women globally, particularly those of reproductive age. It is characterized by a vaginal microbiome with a high overall bacterial density, diverse facultative anaerobes, including *Gardnerella vaginalis*, *Prevotella* spp., *Fannyhessea vaginae*, and *Megasphaera* spp., and few lactobacilli (1). BV is associated with an increased incidence of adverse reproductive outcomes, sexually transmitted infections (STIs), and HIV (2–5). Metronidazole is the usual BV treatment, but despite excellent short-term outcomes over 50% of cases relapse within 12 months (6, 7). BV recurrence is more likely when *Lactobacillus iners* predominates in the vaginal microbiota following treatment, as this species is associated with reduced microbiota stability and a higher likelihood of transition to a BV-type microbiome (8). In contrast, *L. crispatus* is associated with reduced transition to BV-type microbiomes, lower risk of STI/HIV acquisition, and improved reproductive outcomes (9–12).

*Lactobacillus*-based live biotherapeutics offer a promising strategy to sustain an optimal, protective vaginal microbiome after BV treatment. The topical application of a live biotherapeutic product containing a naturally occurring vaginal strain of *Lactobacillus crispatus* CTV-05 (LACTIN-V, Osel Inc.) significantly reduced BV recurrence following metronidazole treatment in a phase 2B clinical trial conducted in the United States (13). However, *L. crispatus* CTV-05 colonization was variable after LACTIN-V application, with three distinct colonization phenotypes based on whether clinically relevant vaginal concentrations of *L. crispatus* (CTV-05 > 10^6^ CFU/mL) were achieved during or after LACTIN-V application (14). Participants who achieved clinically relevant *L. crispatus* CTV-05 colonization during active LACTIN-V application were classified as colonization permissive, whereas those who did not were categorized as colonization resistant (14). In addition, among the colonization permissive group, participants with sustained *L. crispatus* CTV-05 colonization for >3 months after product cessation were termed sustained colonizers, while those who did not were termed transient colonizers (14). Barriers to colonization were also apparent in a more recent clinical trial conducted in young South African women, where LACTIN-V administration increased *L. crispatus* colonization at treatment completion, but where colonization was not sustained at the 4 week follow-up (15). Therefore, barriers to *L. crispatus* colonization are an important knowledge gap. While participants in the US study who were colonization resistant exhibited elevated Shannon diversity prior to LACTIN-V application (14)—suggesting that less effective preceding BV treatment is a barrier—it remains unclear whether adaptive host immune factors might also modulate colonization.

Antibodies play a key role in facilitating local microbial clearance by binding to bacterial surface antigens and activating various immune mechanisms, including opsonization, complement pathways, antibody-dependent cell-mediated cytotoxicity, and agglutination (16). Additionally, antibodies can prevent colonization by immobilizing bacteria and disrupting their adhesion to host epithelial cells (16). Microbe-binding IgA and IgG are present in vaginal secretions, where they are associated with genital inflammation and a reduced vaginal bacterial load, suggesting that they might modulate bacterial colonization of the FGT (17). Therefore, using samples from the U.S.-based trial, we investigated the impact of pre-existing and/or treatment-induced vaginal *L. crispatus* CTV-05-binding antibodies on *L. crispatus* CTV-05 colonization after application of LACTIN-V. Specifically, we hypothesized that vaginal *L. crispatus*-binding antibodies would be induced by the application of the *L. crispatus-*based live biotherapeutic LACTIN-V, and that pre-existing and/or induced host antibody responses would inhibit subsequent colonization efficiency.

## RESULTS

### Study participants

Participants were a subset of a phase 2B randomized, placebo-controlled clinical trial conducted in the United States (13). As previously described (18), for immune analyses we randomly selected 66 participants who had attended all study visits, 32 who received LACTIN-V and 34 who received placebo following metronidazole treatment; the sample from one placebo recipient was damaged in transit, which precluded antibody assays (Table 1). The median age of the remaining 65 participants was 33 years (IQR: 26.5–37.5). Most reported having a male partner within the last 6 months (90.77%), and 35.38% reported using hormonal contraceptives. Study compliance was high, with a median of one missed dose [IQR: 0–2 doses] across both the LACTIN-V and placebo arms. All participants in the LACTIN-V group completed at least 75% of doses, as confirmed by trypan blue staining of intravaginal applicators (13).

**TABLE 1:** Participant characteristics (*n* = 65)^*a*^

Factors	LACTIN-V (*n* = 32)	Placebo (*n* = 33)
Age (yrs)	31.5 [26.25–36.75]	33 [26–38]
Race		
Black	14 (43.75%)	14 (42.42%)
Asian	3 (9.38%)	2 (6.06%)
White	14 (43.75%)	12 (36.36%)
Multiracial	0 (0%)	2 (6.06%)
Unknown	1 (3.13%)	3 (9.09%)
Days since last vaginal sex	4 [3–6.75]	7 [1.5–11.5]
Male partner in the past 6 months	26 (81.25%)	33 (100%)
Female partner in the past 6 months	7 (21.88%)	7 (21.21%)
Douching or intravaginal practices ever	12 (37.50%)	7 (21.21%)
Hormonal contraceptive use	8 (25%)	14 (41.18%)
Number of missed intravaginal doses (total doses: 25)	0.5 [0–2]	1 [0–2.5]

^*a*^ Data are presented as median [IQR] or *n* (%). Information on age, race, sexual history, douching, and hormonal contraceptive use was collected at enrollment (V0).

As previously described (14), based on CTV-05-specific quantitative polymerase chain reaction (qPCR), 23 participants (72%) in the LACTIN-V group were classified as *L. crispatus* CTV-05 colonization permissive, while 9 participants (28%) were colonization resistant. Baseline characteristics did not differ significantly between these groups (Table 1). Among the colonization permissive participants, 13 were classified as sustained colonizers, while 10 were transient colonizers. Sustained colonizers had sex less recently than participants who were colonization resistant (Table S1; *P* = 0.007). The goal in the current study was to assess the relationship between colonization by the *L. crispatus* CTV-05 strain and vaginal CTV-05-binding host antibodies.

### Total and *L. crispatus* CTV-05-binding antibodies in vaginal secretions

Microbe-binding antibody analysis was performed using vaginal swab eluents collected from all study visits for all 32 participants within the LACTIN-V arm and 16 participants within the placebo arm; for the remaining 17 placebo recipients, analysis was limited to the baseline visit with BV (V0) and the post-metronidazole treatment visit (V1). Baseline characteristics did not differ between the two placebo subgroups (Table S1). Detailed demographic data for all participants are presented in Supplemental material S1.

To assess host IgA and IgG binding to the *L. crispatus* CTV-05 strain, we used a previously described flow cytometry-based method, with *L. crispatus*-binding antibody responses reported as an AUC (17) (Fig. S1A). *L. crispatus* CTV-05-binding IgA/IgG antibodies were commonly detected in vaginal secretions, both at baseline and after product application (Table 2). In general, IgA was the predominant CTV-05-binding antibody isotype (Fig. S1B through G), and *L. crispatus* CTV-05-binding IgA and IgG area under the curve (AUC) values correlated with the total vaginal IgA and IgG concentrations (IgA: *r* > 0.377; IgG: *r* > 0.553; all *P* < 0.0001). Overall, *L. crispatus* CTV-05-binding IgA and IgG responses were common but did not correlate with *L. crispatus* CTV-05 abundance, total *L. crispatus* abundance, or total bacterial abundance (16S abundance).

**TABLE 2:** Detectability of *L. crispatus* (CTV-05)-binding IgA and IgG at study visits^*a*^

Time point	*L. crispatus* (CTV-05)-binding IgA	*L. crispatus* (CTV-05)-binding IgG
V0—week 0	46/62 (74.2%)	33/62 (53.2%)
V1—week 1	31/64 (48.4%)	42/64 (65.6%)
V2—week 4	29/47 (61.7%)	31/47 (66.0%)
V3—week 8	35/47 (74.5%)	26/47 (55.3%)
V4—week 12	33/47 (70.2%)	28/47 (59.6%)
V7—week 24	36/47 (76.6%)	34/47 (72.3%)

^*a*^ Data are presented as a count (percentage).

### Pre-treatment *L. crispatus*-binding antibodies do not inhibit colonization after application of an *L. crispatus* live biotherapeutic product

Our first objective was to assess the impact of pre-existing (baseline) vaginal *L. crispatus*-binding IgA and IgG on colonization by *L. crispatus* after LBP application. To investigate this, we assessed total antibody concentration and microbe-binding antibody responses prior to LACTIN-V application, both at baseline (V0) and immediately after BV treatment (V1).

**Fig 1:**
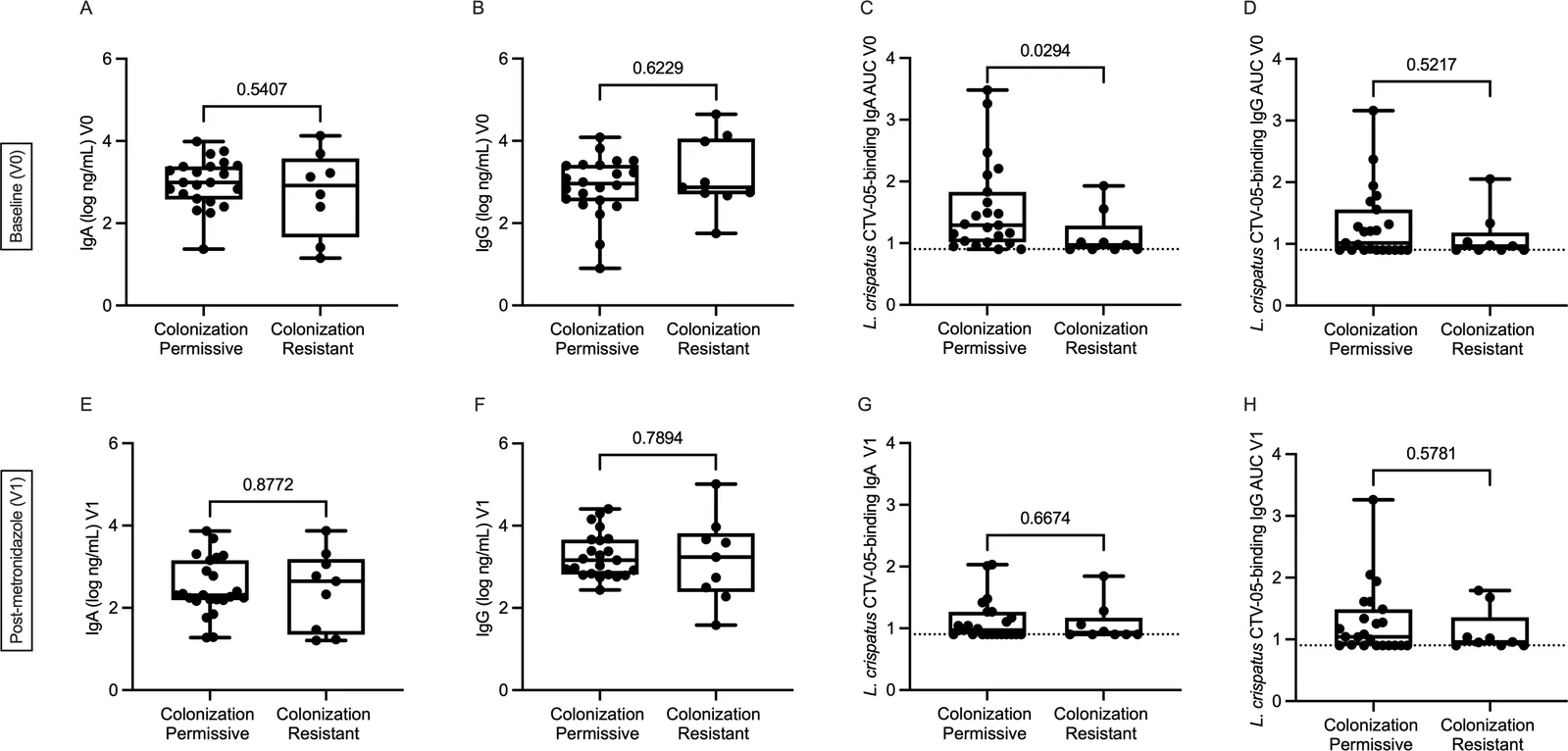
Pre-existing *L. crispatus* CTV-05-binding IgA and IgG and colonization phenotypes. A comparison of (**A–D**) baseline (V0) and (**E–H**) post-metronidazole treatment (V1) concentrations of total vaginal IgA, total vaginal IgG, *L. crispatus* CTV-05-binding IgA, and *L. crispatus* CTV-05-binding IgG between colonization-permissive and colonization-resistant participants. A dotted line denotes the lower limit of quantification (LLOQ) for microbe-binding antibody responses. Statistical analyses include Welch’s T test and Mann-Whitney *U* test.

Contrary to our hypothesis, baseline *L. crispatus* CTV-05-binding IgA responses were significantly higher in colonization-permissive than resistant participants (difference of medians: 0.3217, *P* = 0.0294; Fig. 1A through D). In addition, a higher *L. crispatus* CTV-05-binding IgA response at baseline was associated with both the detection of the *L. crispatus* CTV-05 strain and with total *L. crispatus* abundance at follow-up study visits (V2–V7; all *P* < 0.018). There was no association of colonization phenotype with baseline CTV-05-binding IgG responses, or with either class of CTV-binding antibodies after BV treatment (all *P* > 0.05; Fig. 1E through H).

Overall, 41 participants were BV negative (Nugent score 0–3) at week 12 (V4; LACTIN-V, *n* = 25; placebo, *n* = 16), and 34 remained BV negative at week 24 (V7; LACTIN-V, *n* = 20; placebo, *n* = 14). Participants who remained BV negative following product completion (either LACTIN-V or placebo) exhibited higher baseline *L. crispatus* CTV-05-binding IgA responses (V0; all *P* < 0.05). However, there were no differences in *L. crispatus* CTV-05-binding antibody responses between sustained colonization and transient colonization phenotypes.

### *L. crispatus* CTV-05 antibodies are not induced by LACTIN-V application

**Fig 2:**
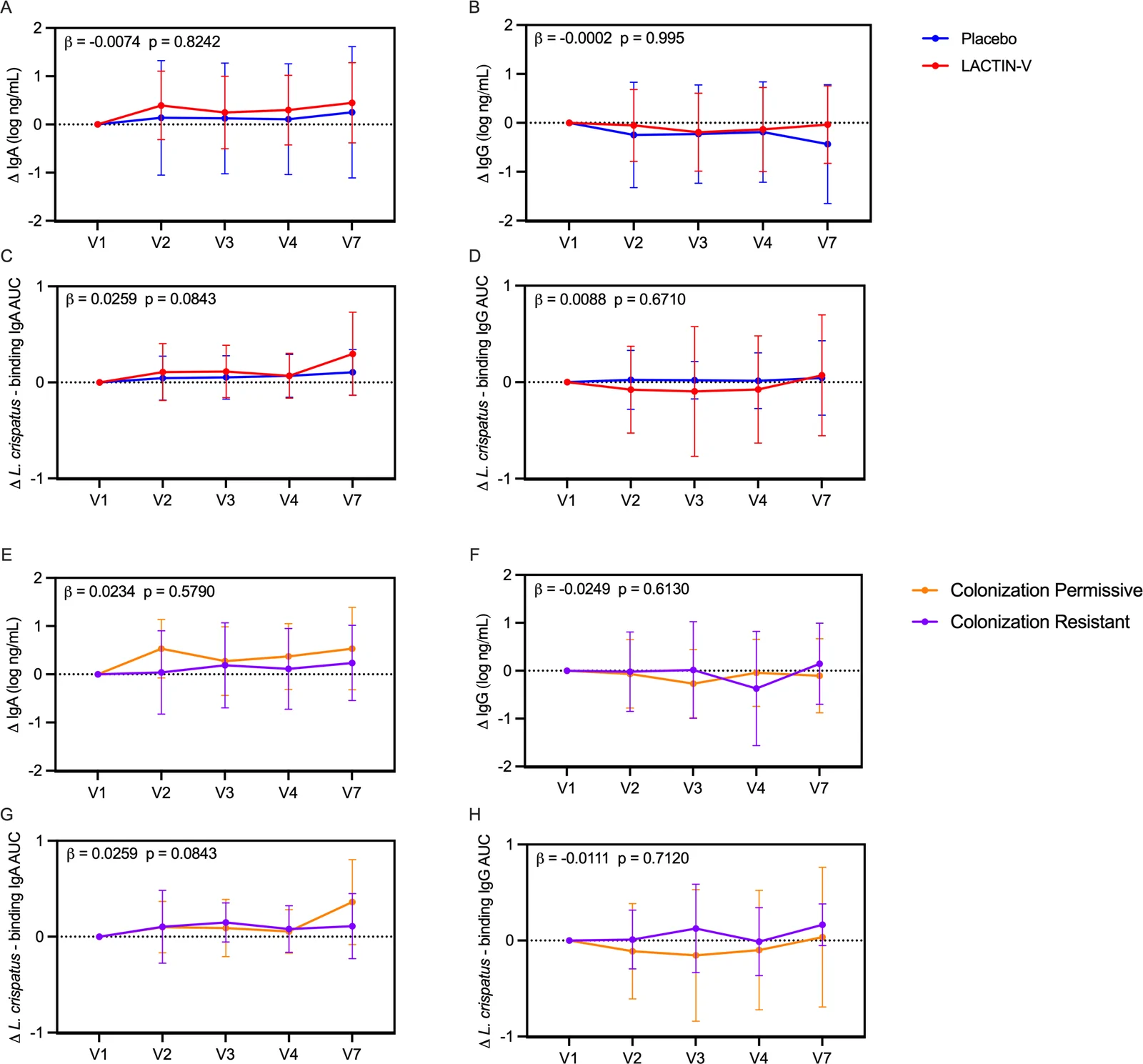
Vaginal IgA and IgG responses following LACTIN-V application. A comparison of the relative change in (**A**) total vaginal IgA, (**B**) total vaginal IgG, (**C**) *L. crispatus*-binding IgA, and (**D**) *L. crispatus*-binding IgG following metronidazole treatment (V1) and the initiation of LACTIN-V or placebo application. A comparison of the relative change in (**E**) total vaginal IgA, (**F**) total vaginal IgG, (**G**) *L. crispatus* CTV-05-binding IgA, and (**H**) *L. crispatus* CTV-05-binding IgG after the initiation of LACTIN-V between colonization-permissive and colonization-resistant participants following metronidazole treatment (V1) and the initiation of LACTIN-V application. The dotted line denotes no change since V1. Statistical analyses are performed using linear mixed-effect models controlling for the random effects of intra-participant variability and adjusting for hormonal contraception and vaginal douching. The β estimates (*P* value) depict the interaction between time and LACTIN-V treatment and colonization-permissive phenotypes, respectively.

Our second objective was to assess whether LACTIN-V application induced changes in vaginal antibody responses over time, which we assessed using linear mixed-effects models (see Materials and Methods). We found that total IgA concentrations increased modestly over time in all participants (β = 0.061, SE = 0.027, *P* = 0.025), but there was no evidence that LACTIN-V application induced a change in either total vaginal IgA/IgG concentrations or in *L. crispatus* CTV-05-binding IgA/IgG responses over time when compared to placebo (time × treatment interactions: IgA: β = −0.007, SE = 0.034, *P* = 0.825; Fig. 2A through D). Next, we performed a sensitivity analysis using a restricted model in which we only included study visits during active LACTIN-V application and the visit immediately following LACTIN-V completion (V4), as any antibody production by week 24 (13 weeks off product) is unlikely to be due to LACTIN-V application. Again, LACTIN-V application did not result in a change in vaginal IgA/IgG concentrations nor *L. crispatus* CTV-05-binding IgA/IgG responses over time. Hormonal contraception use and douching were not significantly associated with CTV-05-binding antibodies. Given the absence of interaction between time and LACTIN-V application, additional post hoc pairwise comparisons were not pursued.

Using similar linear mixed-effects models, we next investigated whether specific *L. crispatus* CTV-05 colonization phenotypes were associated with changes in vaginal antibody responses. We observed a modest increase in *L. crispatus* CTV-05-binding IgA responses in colonization-permissive participants compared to colonization-resistant participants (β = 0.0433, SE = 0.0213, *P* = 0.0439). However, in the restricted model, the interaction between colonization permissiveness and time was no longer statistically significant (β = −0.013, SE = 0.038, *P* = 0.73). Neither model found that colonization permissiveness was associated with differences in *L. crispatus* CTV-05-binding antibody responses or total vaginal IgA/IgG concentrations over time (Fig. 2E through H), and neither was sustained versus transient colonization associated with antibody changes over time (all *P* > 0.05; Fig. S2) in either model.

Missed product doses were infrequent in both the LACTIN-V and placebo groups (Table 1), and we observed no correlation between the number of missed LACTIN-V doses and vaginal mucosal IgA or IgG levels.

## DISCUSSION

LACTIN-V, a live biotherapeutic product containing the *Lactobacillus crispatus* strain CTV-05, promotes vaginal *L. crispatus* colonization and reduces BV recurrence rates following the treatment of clinical BV (13). However, rates of *L. crispatus* CTV-05 colonization after LACTIN-V application are variable, with only a subset of participants achieving and sustaining *L. crispatus* CTV-05 colonization (14, 15). Since vaginal microbe-binding antibodies have been associated with reduced bacterial abundance and enhanced vaginal inflammation (17), in this study we aimed to assess whether pre-existing or induced vaginal antibodies binding the *L. crispatus* live biotherapeutic strain CTV-05 might modulate *L. crispatus* colonization. We demonstrate that vaginal *L. crispatus* CTV-05-binding IgA and IgG were common, with IgA being the dominant binding isotype. However, neither pre-existing vaginal *L. crispatus* CTV-05-binding IgA nor IgG hindered subsequent CTV-05 colonization; indeed, elevated baseline *L. crispatus* CTV-05-binding IgA was unexpectedly associated with enhanced CTV-05 colonization, although this finding will require confirmation in future studies. Furthermore, LACTIN-V application did not induce vaginal CTV-05-binding IgA or IgG antibodies, regardless of subsequent colonization patterns.

Identifying barriers to sustained colonization is critical to optimizing the efficacy of live biotherapeutic products, such as LACTIN-V. Since our group and Schuster et al. independently demonstrated lower microbe-binding IgA and IgG levels among women with more diverse vaginal microbiomes, such as BV (17, 19, 20), we hypothesized that the pre-application presence of host vaginal antibodies able to bind the active bacterial ingredient in the LBP (i.e., *L. crispatus* CTV-05) would inhibit colonization. However, our findings indicate that pre-existing *L. crispatus* CTV-05-binding antibody responses did not prevent *L. crispatus* colonization, and that pre-existing IgA responses were actually associated with enhanced colonization. While the reason for this is not clear, we hypothesize that the presence of these responses may reflect preceding colonization by *L. crispatus* before the onset of BV, therefore representing a biomarker for a vaginal environment that is more permissive to *L. crispatus*. However, since pre-enrollment microbiota data were not available, we are unable to directly test this hypothesis. Alternatively, IgA might directly facilitate bacterial colonization by promoting adherence to the epithelium, as has been demonstrated in the GI tract (21). If future studies confirm our findings, it will be interesting to assess whether vaginal IgA plays an active role in promoting *L. crispatus* colonization or is merely a marker of a mucosal environment that is conducive to colonization.

Importantly, we did not find any evidence that LACTIN-V application induces vaginal mucosal IgA or IgG responses to the *L. crispatus* CTV-05 strain, regardless of baseline humoral immune parameters or colonization phenotype. This lack of mucosal antibody induction by LACTIN-V may reflect prior exposure to *L. crispatus* with subsequent immune tolerance, low immunogenicity of the CTV-05 strain, or the absence of vaginal immune-inductive sites (mucosa-associated lymphoid tissues). This is reassuring, since both microbe-binding IgA/IgG and total vaginal concentrations of IgA and IgG were associated with vaginal epithelial disruption and inflammatory immune factors linked to HIV acquisition in previous studies (22). However, this should be confirmed through future studies investigating the immune effects of extended *L. crispatus* LBP use and/or repeated cycles of LBP administration.

Our study has several limitations. First, our assay only used the *L. crispatus* CTV-05 strain to detect microbe-binding antibody responses. While this maximized our ability to assess responses to the strain utilized in the trial-administered LBP, there is the potential for strain specificity of *L. crispatus*-binding antibodies, and so it would be interesting for future studies to assess a wider panel of *L. crispatus* strains. An inherent limitation of our assay is that it cannot quantify antibodies that had already been bound by endogenous vaginal bacteria, and so would not be present in vaginal secretions, as previously noted (17). While the prospective clinical trial design was a strength, several potential confounders could not be assessed since data had not been collected regarding parameters, such as stage of the menstrual cycle. Finally, the relatively small sample size limited our statistical power to detect subtle differences in antibody responses across colonization phenotypes, or to assess potentially important impacts of parameters, such as hormonal contraception or sexual intercourse. Accordingly, these findings should be interpreted as hypothesis-generating and warrant validation in larger, independent studies.

In summary, we demonstrate that pre-existing *L. crispatus* CTV-05-binding IgA and IgG do not inhibit vaginal *L. crispatus* colonization after application of a vaginal live biotherapeutic, and that *L. crispatus* CTV-05-binding IgA was associated with colonization permissiveness. Furthermore, LACTIN-V application did not induce *L. crispatus* CTV-05-binding IgA or IgG responses, or alter total vaginal IgA and IgG concentrations.

## MATERIALS AND METHODS

### Study participants

Female participants were recruited into a previously described phase 2b randomized placebo-controlled clinical trial of the *L. crispatus*-based live biotherapeutic (LACTIN-V) to prevent BV recurrence following standard antibiotic treatment (13). Briefly, women with BV (as defined by ≥3 Amsel criteria and a Nugent score ≥4) at baseline visit (V0) were given a 5 day course of topical metronidazole and then randomized 2:1 to either LACTIN-V or placebo treatment arms. Treatments were self-administered intravaginally once daily for 5 days, followed by twice weekly for 10 weeks. Vaginal swabs were collected at baseline (V0) and within 48 h of completing metronidazole treatment (V1; 7 days after baseline) and at 4 weeks (V2), 8 weeks (V3), 12 weeks (V4; 1 week after the last dose of LACTIN-V), and 24 weeks (V7; 13 weeks after the last dose of LACTIN-V) after enrollment. Immediately following collection, vaginal swabs were plunged into 2 mL of Starplex transport medium, immediately frozen at −20°C, and then transferred to a −80°C freezer at the end of the day. Of the 228 participants enrolled in the parent clinical trial, 66 were randomly selected by a third-party data management group (EMMES) in a 1:1 ratio of LACTIN-V and placebo recipients. Eligible participants were required to have attended all scheduled study visits with available samples and to have demonstrated ≥75% treatment compliance, thereby representing a highly adherent subset. One sample from a participant in the placebo group was damaged during transport and was therefore excluded from analysis. The final analytical cohort therefore consisted of 65 highly adherent participants, including 32 randomized to the LACTIN-V treatment arm and 33 randomized to placebo.

### Sex as a biological variable

This study exclusively included female participants, since BV is a condition specific to the vagina. Data regarding contraceptive use and sexual intercourse, but not menstrual cycle phase, were collected during the clinical trial and included in relevant analyses.

### Total IgA and IgG quantification

Vaginal swab supernatants were diluted in PBS with 1% Tween 20 and 10% BSA and centrifuged at 16,000 × *g* for 10 min. Total IgA and IgG levels were quantified using human IgA and IgG (total) uncoated ELISA kits (Invitrogen) according to the manufacturer’s instructions. The lower limit of quantification (LLOQ) for each assay was defined as the highest LLOQ across all experiments as determined by the standard curve, and results below this were assigned the LLOQ value. Internal laboratory controls were used as inter-assay controls.

### Microbe-binding assay

The anti-commensal antibody protocol was adapted from Moor et al. (23) as previously described (17). Undiluted vaginal swabs were heat-inactivated for 30 min at 56°C and serially diluted in FACs buffer (1:2 dilutions in FACs [2% BSA, 5 µM EDTA-PBS]). Equal volumes of vaginal samples were combined with an equal volume of bacteria (~10^5^ CFU/mL) and incubated for 1 h at 4°C. Antibody-bound bacteria were stained with anti-human goat IgG F(ab′)2 antibody (AF594, Jackson Immunology Inc.) and anti-human goat IgA F(ab′)2 antibody (AF647, Southern Biotech). All samples were analyzed using an LSR Fortessa (Becton Dickinson) with settings optimized for bacterial cell detection. A sample of bacteria stained with secondary antibodies in the absence of any clinical samples was used as a negative control and subsequently subtracted from all clinical samples to control for non-specific staining. Flow cytometry analysis of samples was performed using FlowJo (Treestart Inc.), with fluoresce-minus-ones and negative controls used to determine the gating strategies; representative gating strategies have been previously presented elsewhere (17). Geometric mean fluorescence intensity (MFI) values were extracted for each sample at each dilution. Sample dilution factors and their corresponding MFI values were log10 transformed and plotted with a linear regression using GraphPad Prism to produce regression lines for each sample. The MFI LLOQ value was set as 10; all values below the LLOQ were assigned the LLOQ value, as only values above 10 were reproducible across experiments. Responses were reported as the AUC value for each regression line. IgG+ and IgA+ events were reported from the highest concentration dilution of each vaginal sample.

### Bacterial preparations

*L. crispatus* (CTV-05; OSEL) was grown in MRS agar anaerobically (80% N_2_; 10% CO_2_; 10% H_2_) at 37°C for 24 h. Bacteria were harvested at a concentration of ~1 × 10^9^ CFU. Bacteria were stained with CFSE (Thermofisher) for 30 min and washed before incubation with clinical samples.

### DNA extraction and quantitative polymerase chain reaction

DNA was extracted from 175 μL of the bacterial pellet in vaginal swab samples using the Qiagen DNeasy PowerSoil kit (Qiagen). Targeted quantitative polymerase chain reaction was used to estimate total bacterial abundance by targeting the 16S region of the rRNA gene, and the absolute abundances of *Lactobacillus* species (*L. crispatus*, *L. iners*, *L. jensenii*, and *L. gasseri*) using a multiplex assay with previously validated primer and probe sequences. All quantitative polymerase chain reaction assays were TaqMan-based and performed on the QuantStudio 6 Flex Real-Time PCR System (Thermofisher). A no-template control containing nuclease-free water was included in all qPCR experiments to monitor for contamination and non-specific amplification. Total bacterial load was quantified with qPCR adopted from Nadkarni et al. (24). Protocol for quantification of *L. crispatus*, *L. iners*, *L. jensenii*, and *L. gasseri* absolute abundances with multiplex quantitative polymerase chain reaction was adopted from Balashov et al. (25). Total reaction volume for assays was 10 μL. Assays for *L. crispatus*, *L. iners*, *L. jensenii*, and *L. gasseri* were performed at 95°C for 10 min, 45 cycles at 95°C for 15 s then 60°C for 1 min. Data analysis was performed with QuantStudio Real-Time PCR Software version 1.3 (Applied Biosystems). Copy numbers were quantified using the following equation, where ΔCt represents the difference in Ct between a sample and negative control: $Copy\;number=2^{\Delta Ct}$.

Polymerase chain reactions specific for the *L. crispatus* CTV-05 strain were performed at the University of California, San Francisco, using gene targets for strain and species-specific PCR identified in *L. crispatus* CTV-05-specific regions which were absent in other sequenced bacterial strains and with low numbers of homologs in vaginal metagenome data sets, as previously described in detail (14); the limit of detection at the 95% detection threshold was 6.0 × 10^2^ CFU/mL. A concentration of *L. crispatus* CTV-05 greater than 10^6^ CFU/mL was pre-defined as the threshold for clinically relevant colonization, based on published evidence that this concentration of *L. crispatus* CTV-05 was associated with reduced recurrence of BV (13) and UTIs (26, 27).

### Statistical analysis

Analysis was performed using either GraphPad Prism (version 10.1.0) or RStudio (version 2022.07.02). Bacterial abundance and antibody concentrations were log10 transformed for all analyses. Linear mixed-effects models were created using the lmertest R package (version 3.1.3). To assess changes in vaginal antibody responses over time, we fitted linear mixed-effects models with participant-specific random intercepts to account for repeated measures within individuals. Fixed effects included time, treatment group, their interaction (time × treatment), and covariates for hormonal contraception use and douching. The interaction term was used to test whether IgA trajectories differed between LACTIN-V and placebo groups after adjustment for these covariates.

## Data Availability

All metagenomic sequencing data are available on the NCBI Sequence Read Archive under accession numbers PRJNA784288 and PRJNA1090602.

## SUPPLEMENTAL MATERIAL

Supplemental tables and figures spectrum.03983-25-s0001.docx Tables S1 and S2, and Fig. S1 to S3.

Supplemental materials spectrum.03983-25-s0002.xlsx Detailed demographics.

ASM does not own the copyrights to Supplemental Material that may be linked to, or accessed through, an article. The authors have granted ASM a non-exclusive, world-wide license to publish the Supplemental Material files. Please contact the corresponding author directly for reuse.
