# Supplementary material for: Vaginal microbe-binding antibody interactions with a Lactobacillus crispatus live biotherapeutic following treatment for bacterial vaginosis
Source: Microbiol Spectr. 2026 Sep 1;14(10):e03983-25. doi: 10.1128/spectrum.03983-25 (PMC13689376; doi:10.1128/spectrum.03983-25)
Supplement: Supplementary file 1 [file spectrum.03983-25-s0001.docx]

**Supplementary Tables and Figures**

**Supplementary Table 1. Characteristics of the sub-groups of participants that received either LACTIN-V (n=32) or Placebo (n=33).**

| Factors | LACTIN-V (n=32) | | | Placebo (33) | |
| --- | --- | --- | --- | --- | --- |
|  | Sustained colonizers (n=10) | Transient colonizers (n=13) | Colonization resistant (n=9) | Placebo subset 1 (n=16)  *All timepoint analyzed* | Placebo subset 2 (n=17)  *Only V0 and V1 analyzed.* |
| Age (yrs) | 30 [26.5-33.25] | 33 [25-37.5] | 33 [25-45] | 30.5 [22-35] | 37 [30-39.5] |
| Race  Black  Asian  White  Multiracial  Unknown | 2 (20%)  2 (20%)  6 (60%)  0 (0%)  0 (0%) | 6 (46.15%)  1 (7.69%)  6 (46.15%)  0 (0%)  0 (0%) | 6 (66.67%)  0 (0%)  2 (22.22%)  0 (0%)  1 (11.11%) | 6 (37.50%)  0 (0%)  6 (37.50%)  1 (6.25%)  3 (18.75%) | 8 (47.06%)  2 (11.76%)  6 (35.29%)  1 (5.88%)  0 (0%) |
| Days since last vaginal sex | 7 [6-12.25]** | 4 [2.5-5.5] | 3 [1-3]** | 7.5 [1-14] | 7 [2-10] |
| Male partner in the past 6 months | 8 (80%) | 9 (69.23%) | 9 (100%) | 16 (100%) | 17 (100%) |
| Female partner in the past 6 months | 4 (40%) | 3 (23.08%) | 0 (0%) | 4 (25%) | 3 (17.65%) |
| Douching or intravaginal practices ever | 3 (30%) | 5 (38.46%) | 4 (44.44%) | 5 (31.25%) | 3 (17.65%) |
| Hormonal contraceptive use | 2 (20%) | 3 (23.08%) | 3 (33.33%) | 7 (43.75) | 7 (41.18%) |
| Number of missed intravaginal doses | 0.5 [0-2.25] | 0 [0-1.5] | 0 [0-3.5] | 1 [0-2] | 0 [0-3.5] |

Data are presented as median [IQR] or n (%). Information on age, race, sexual history, douching, and hormonal contraceptive use were collected at enrollment (V0). Statistical comparisons are performed using one-way ANOVA with Tukey’s post hoc, Kruskal-Walis test with Dunn’s post hoc, Welch’s T-test, Fisher’s exact test, and the Man-Whitney U test between sub-groups within the same treatment arm. Statistical differences are denoted between groups using * p<0.05, **p<0.01, ***p<0.001, ****p<0.0001

**Supplemental Table 2. Detectability of *L. crispatus*-(CTV-05) binding IgA and IgG by treatment group.**

|  | *L. crispatus* (CTV-05) – binding IgA | | *L. crispatus* (CTV-05) – binding IgG | |
| --- | --- | --- | --- | --- |
| Timepoint | LACTIN-V treated | Placebo treated | LACTIN-V treated | Placebo treated |
| V0 – Week 0 | 26/32 (81.25%) | 21/31 (67.74%) | 19/32 (59.38%) | 14/31 (45.16%) |
| V1 – Week 1 | 16/32 (50%) | 13/30 (43.33) | 31/32 (96.88%) | 20/30 (66.67%) |
| V2 – Week 4 | 20/32 (62.5%) | 9/15 (60%) | 22/32 (68.75%) | 9/15 (59.34%) |
| V3 – Week 8 | 24/32 (75%) | 11/15 (73.33%) | 18/32 (56.25%) | 8/15 (53.33%) |
| V4 – Week 12 | 23/32 (71.88%) | 10/15 (33.33%) | 19/32 (59.34%) | 9/15 (59.34%) |
| V7 – Week 24 | 24/32 (75%) | 12/15 (80%) | 24/32 (75%) | 10/15 (66.67%) |

**Supplementary Figure 1. *L. crispatus* CTV-05 binding IgA and IgG responses in vaginal swabs. (A)** Representative flow cytometry dot plots and histograms of *L. crispatus* CTV-05 binding IgA vs. IgG responses within vaginal swabs from the LACTIN-V clinical trial cohort. The four overlaid colours represent the four dilutions analyzed for each clinical sample (length on the left). **(B-G)** A comparison of the total IgA+ proportion and the total IgG+ proportion at each time point. Statistical analyses are performed using Wilcoxon’s test.

**Supplementary Figure 2. Pre-existing *L. crispatus* - binding IgA and IgG and colonization phenotypes.** A comparison of baseline (V0; **A-D**) and post-metronidazole treatment (V1; **E-H**) concentrations of total vaginal IgA, total vaginal IgG, *L. crispatus* CTV-05-binding IgA, and *L. crispatus* CTV-05-binding IgG between colonization resistant, transient colonizers and sustained colonizers. The dotted line denotes the LLOQ of microbe-binding antibody responses. Statistical analyses include Kruskal-Walis test with Dunn’s post hoc test and one-way ANOVA with Tukey’s post hoc test.

**Supplementary Figure 3. Changes in vaginal IgA and IgG responses between colonization phenotypes following LACTIN-V application.** A comparison of the relative change in **(A-D)** total vaginal IgA, **(E-H)** total vaginal IgG, **(I-L)** *L. crispatus* CTV-05-binding IgA, and **(M-P)** *L. crispatus* CTV-05-binding IgG following metronidazole treatment (V1) to visits following the initiation of LACTIN-V application between colonization resistant, transient colonizers, and sustained colonizers. The dotted line denotes no change since V1. Statistical analyses are performed using the Kruskal-Wallis test with Dunn’s post hoc test.

**Supplementary Material 1. Extended participant demographics table.**
